# Advances in Plant-Derived Scaffold Proteins

**DOI:** 10.3389/fpls.2020.00122

**Published:** 2020-02-25

**Authors:** Congyue Annie Peng, Lukasz Kozubowski, William R. Marcotte

**Affiliations:** Department of Genetics and Biochemistry, Clemson University, Clemson, SC, United States

**Keywords:** spider silk, collagen, elastin, scaffold, regenerative medicine, extracellular matrix

## Abstract

Scaffold proteins form critical biomatrices that support cell adhesion and proliferation for regenerative medicine and drug screening. The increasing demand for such applications urges solutions for cost effective and sustainable supplies of hypoallergenic and biocompatible scaffold proteins. Here, we summarize recent efforts in obtaining plant-derived biosynthetic spider silk analogue and the extracellular matrix protein, collagen. Both proteins are composed of a large number of tandem block repeats, which makes production in bacterial hosts challenging. Furthermore, post-translational modification of collagen is essential for its function which requires co-transformation of multiple copies of human prolyl 4-hydroxylase. We discuss our perspectives on how the GAANTRY system could potentially assist the production of native-sized spider dragline silk proteins and prolyl hydroxylated collagen. The potential of recombinant scaffold proteins in drug delivery and drug discovery is also addressed.

## Introduction

Scaffold proteins, synthetic or natural, provide promising innovative solutions to regenerative medicine (Lavine et al., 2012; Bourzac, 2015; Gould, 2015). A broad range of functional proteins with superb biocompatibility and biodegradability such as helical collagen (Parry et al., 2005), elastin (Mithieux and Weiss, 2005), silkworm silks (Dicko et al., 2006), and spider silks (Gosline et al., 1999) are candidates for proteinaceous scaffold biomaterials. The source of proteins to be processed to biomaterial scaffolds is often limited, unsustainable, or sometimes carries the risks of human pathogen contamination (Wong Po Foo and Kaplan, 2002). Recombinant production systems, such as mammalian cells, insect cells, silkworms, yeast (*Pichia pastoris*), *Escherichia coli*, and plants provide opportunities to produce scaffold proteins in full length or as representative motifs. Recombinant production of scaffold proteins also allows the flexibility of engineering variants or combinations of motifs that are difficult or impossible to obtain from natural sources. Each system has gained some successes, but the intrinsic nature of the host systems and economic feasibility are often a source of limitations.

Plant host systems demand less in energy input, chemical reagents, and contamination management (Shoseyov et al., 2014). In theory, sustainable and cost-effective production of recombinant scaffold proteins are feasible using plant hosts (Scheller and Conrad, 2005). Depending on the recombinant proteins, eukaryotic post-translational modifications can presumably be achieved inside plant cells as the proteins mature (Ruggiero et al., 2000). This review summarizes the advances and challenges in plant host-derived scaffold proteins: spider silk protein, collagen, elastin, and a bone matrix protein, with the focus on fusion tags that facilitate protein accumulation in the endoplasmic reticulum (ER) and subsequent protein purification. Techniques with the potential to improve yield to meet the requirement of economical application, methods allowing efficient transformation of several genes or large repeat domains, and the utilization of scaffold proteins for drug screening are also discussed.

## Spider Silk-Based Scaffold Protein Produced From Plants

### Major Ampullate Spidroin Sequence, Structure and Folding

Both spider silk and silkworm silks are superior materials for tissue regeneration (Holland et al., 2019). The abundant availability of silkworm silk extracted from silkworm cocoons permits its extensive application in tissue engineering through materials fabrication (Huang et al., 2018). Purification of silkworm silks requires a degumming process to remove sericin, a protein component in the silkworm cocoon and the outer layer of the silkworm silks that stimulates immune response (Altman et al., 2003). Spider dragline silk, the life-line of an orb-weaving spider, has impressive mechanical strength and toughness, which makes it a potential candidate for applications such as bone regeneration (Gosline et al., 1999; Gomes et al., 2011; Andersson et al., 2016; Lee M. et al., 2016). In addition, spider silk propagates light as an optical fiber (Huby et al., 2013). Although the cannibalistic nature of spiders precludes the possibility of farming (Nentwig, 2013), recombinant production of spider silk proteins provides alternatives to obtain spider silk-like proteins. Recombinant production also allows generation of biomaterials in a diverse range of forms, such as hydrogels, films, coatings, meshes, and nanoscale particles and tubes.

#### Major Ampullate Spidroin Types

**Figure 1:**
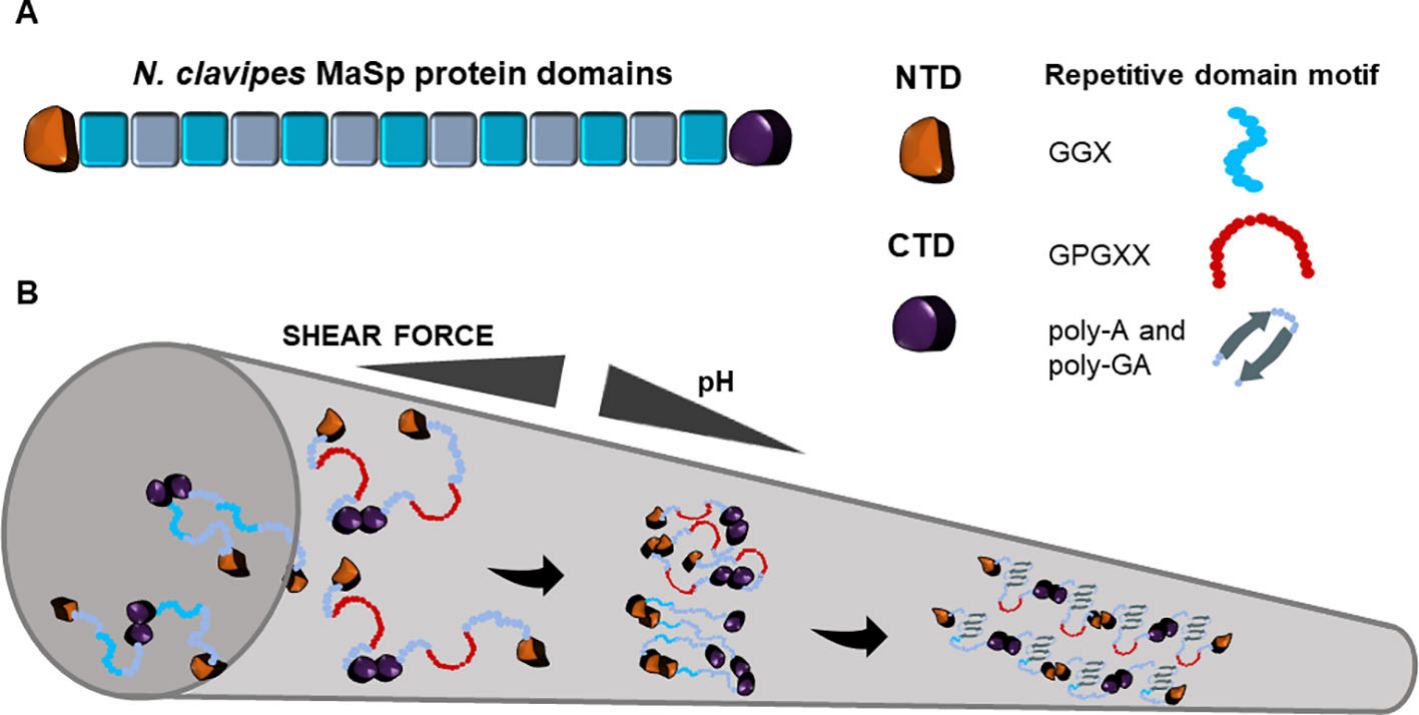
Schematic representation of Nephila clavipes major ampullate spidroin domains **(A)** and the fiber assembly model in major ampullate spinning duct **(B)**. The colored blocks represent repeat blocks with the color variation indicating the glycine-rich motif and the poly-alanine motif. Along the tapered spinning duct, with the increase of shear force and the decrease of pH, *N. clavipes* major ampullate spidroins transit from the liquid unfolded state into oligomeric state through the dimerization of N-terminus and the facilitation of C-terminus. The oligomeric state ultimately transitions into a two-phased state, which is comprised of unordered amorphous regions and ordered β-sheet crystalline regions.

Two major spidron proteins were identified from *Nephila clavipes* spider dragline silk, namely major ampullate spidroins 1 and 2 (MaSp1, MaSp2). Each protein contains conserved tandem block repeats and the flanking non-repetitive N- and C- terminal domains that are conserved in all orb-weaving spiders (Xu and Lewis, 1990; Hinman et al., 1992, **Figure 1**). The predicted 3.45 Gb genome of *N. clavipes* encodes eight potential MaSps, ranging in sizes from ~100 to over 3,000 amino acids with motifs mapped to the originally discovered MaSps. To differentiate MaSps discovered from the genome assembly, the new nomenclature set of MaSp-a through MaSp-h is used (Babb et al., 2017). Studies prior to the genome assembly have used the previous nomenclature: MaSp1 and MaSp2. MaSps are diverse but some motifs are conserved among arachnid species (Gatesy et al., 2001). Proteotranscriptomic study of the *N. clavipes* major ampullate gland detected transcripts and protein products of the two primary spidroins: *MaSp1* and *MaSp2*. Importantly, proteins involved in ion transport, folding and conformation regulation, post-translational modification, and fibrillar preservation and protection were simultaneously detected, indicating the complexity of spidroin production and the following transition into solid fibers (Santos-Pinto et al., 2019). Despite similar domain structures, the isoelectric point of the two spidroins differs. Isoelectric focusing analysis indicate the p*I* of MaSp1 is above 8.5 and the p*I* of MaSp2 is between 5.1 and 5.9 (Sponner et al., 2005a).

#### Major Ampullate Spidroin Repeat Domain

The tandem block repeat domains of MaSp1 and MaSp2 differ slightly from each other but can be generalized into the following consensus sequences (Hayashi et al., 1999):

MaSp1: (GGA) GQ (GGY) (GGL) (GGQ) GAGR (GGL) (GGQ) (GA)_2_ (A)_3_
MaSp2: (GPGGY) (GPGQQ) (GPGGY) (GPGQQ) GPSGPS (A)_9_

The ~100 block repeats cover about 95% of each protein sequence. The main difference between MaSp1 and MaSp2 is that MaSp1 repeat region is essentially devoid of proline. Proline is believed to contribute to the major structural and mechanical difference between MaSp1 and MaSp2 (Marhabaie et al., 2014). The proline kink in the polypeptide backbone causes a reduction in hydrogen bond donor capacity and adversely affects β-sheet extension of the poly alanine residues.

#### Major Ampullate Spidroin C-Terminal Domain (CTD)

The short non-repetitive C-termini of MaSp1 and MaSp2 share 75% identity (Beckwitt and Arcidiacono, 1994). Three conserved sites were identified between *N. clavipes* MaSp and minor ampullate spidroin (MiSp) CTDs (Collin et al., 2018). The CTD folds into five parallel helices and forms a homodimer stabilized through a disulfide bond between conserved cysteine residues and two salt bridges (Hagn et al., 2010). *In vitro* experiments show that recombinant CTDs attached to the repeat domain lead to faster aggregation of the repeat domain and are more susceptible to buffer-induced fibril assembly (Huemmerich et al., 2004a; Huemmerich et al., 2004b; Sponner et al., 2005b; Hedhammar et al., 2008). The CTD reinforces protein alignment, exposes its hydrophobic region to attract CO_2_ for binding, and facilitates β-amyloid like nucleation (Ittah et al., 2006; Eisoldt et al., 2010; Andersson et al., 2014).

#### Major Ampullate Spidroin N-Terminal Domain (NTD)

The non-repetitive, hydrophilic NTD is conserved among species, within different types of spider silk proteins (dragline, flagelliform, and cylindriform silk), and among waxmoth and silkworm fibroins (Bini et al., 2004; Motriuk-Smith et al., 2005; Rising et al., 2006; Collin et al., 2018). In spun dragline silk, the NTD can be detected in both inner and outer core regions (Andersson et al., 2013), indicating it is not being cleaved in the process of fiber assembly. MaSp1 NTD is composed of five anti-parallel α-helices, forms thermally stable homodimers at pH 6, and restricts CO_2_ binding (Askarieh et al., 2010; Hagn et al., 2011; Andersson et al., 2014). Asymmetric interaction of amino acid residues and salt bridge formation is detected and is believed to contribute to the plasticity of the *N. clavipes* NTD (Atkison et al., 2016). Folding at the area surrounding a conserved tryptophan residue in the stable NTD homodimer compels the downstream repeat domains to initiate β-sheet orientation (Askarieh et al., 2010; Gaines et al., 2010). NTD motifs, essential for folding and response to acidic pH, are structurally homologous between species (Heiby et al., 2017). The dipole-dipole interaction of NTD monomers at acidic pH may reduce the free energy barrier required to initiate dimer association and become the driving force for fiber assembly (Ries et al., 2014: Barroso da Silva et al., 2016).

#### Major Ampullate Spidroin Post-Translational Modification (PTM)

Major ampullate spidroins obtained from dragline silk fibers and gland extractions contain post-translational modifications, such as tyrosine or serine phosphorylation (Michal et al., 1996). L-Dopa (3,4-dihydroxyphenylalanine) and dityrosine are detected from hydrolyzed major dragline silk solutions, but it is not clear if this result may be the result of tyrosine oxidation during sample preparation or the exposure of fibers post-spinning to uv radiation (Santos-Pinto et al., 2014). Phosphorylation sites found in MaSp1 isoforms A and B reside almost exclusively in the GGX region of the repeat domain (Santos-Pinto et al., 2015). For MaSp2, phosphorylation sites are found both in the repeat domain and C-terminus. The phosphorylation sites of MaSp2 repeat domain reside at identical positions within each repeat domain (Santos-Pinto et al., 2016). The reason why only certain locations in the repeat domain are phosphorylated is unknown. We speculate that these special locations may reflect the inter- and intra-molecular interaction in the 3D structure after protein folding, through which a hierarchical network can be formed and eventually lead to the fiber assembly.

*N. plumipes* dragline silk displays an arginine hydrogen bonding with the amorphous regions, and hydroxyproline is detected through Dynamic Nuclear Polarization (DNP) NMR spectroscopy (Craig et al., 2019). Peptide glycosylation is detected in the solubilized dragline silk of *N. clavipes*, although the oligosaccharide molecule is still unknown (Guehrs et al., 2008). While PTM may play a critical role in fiber assembly, the mechanisms of PTM of spidroins in spider ampullate gland are poorly understood. The recent proteomic data of the gland may shed some light on the mechanism involved in PTM of spidroins (Santos-Pinto et al., 2019).

#### Molecular Basis of Major Ampullate Spidroin Biomaterials Assembly

The transition from liquid crystalline folding of spidroins to solid fibers in the spider duct is a complex process that is far from being understood (Vollrath, 2016). The current model on spidroin fiber assembly is shown in **Figure 1**. Progress has been made to capture the fiber assembly initiator structures such as transient oligomers (Landreh et al., 2017) or oligomeric micelles (Bauer and Scheibel, 2017), providing evidence that fiber assembly may involve formation of initial “seed” scaffolds for further nucleation of additional spidroins. The transition requires 300–700 MPa shear force and at least 6 poly alanine modules (Giesa et al., 2016). In the solid fiber state, 40% of alanine forms oriented β-sheet stacked crystallites and about 60% of alanine folds into poorly oriented β-sheet (Simmons et al., 1996; Ene et al., 2009). The ordered and amorphous regions provide the molecular basis that allows physical strength and extensibility in one fiber (Patil et al., 2014; Xu et al., 2014). This two-phased structural arrangement also contributes to phonon propagation bandgap along the fiber axis (Schneider et al., 2016).

### Plant-Derived Spidroins

Recombinant spidroin analogs have provided useful testing materials for *in vitro* assessment of issues such as protein solubility and/or premature aggregation, translational pause, and post-translational modification. Host system selection, however, impacts the quality and quantity of the recombinant protein production. Each host system offers promising possibilities but possesses also some limitations.

To attain the desired mechanical properties, recombinant major ampullate spidroin proteins (rMaSp1 and rMaSp2) are expected to contain large numbers of repeat domains, ideally equivalent to the native protein (~100 repeat domains) and produce a large protein with molecular weight greater than 300 kDa. Engineering ~100 repeat domains is a challenge for cloning, protein solubility during purification, and host transcriptional regulation (Guerette et al., 1996; Lewis et al., 1996; Fukushima, 1998; Xia et al., 2010; Hauptmann et al., 2013). The frequent demand for alanine and glycine depletes the correspondent tRNA pools quickly and causes translational pause (Candelas et al., 1990). With the advances in function elucidation of the non-repetitive terminal domains, many efforts are devoted to incorporation of native N- and C-termini in recombinant spidroins to enhance solubility, protein alignment, and fibril assembly (Ittah et al., 2006; Barroso da Silva et al., 2016). Efforts to functionalize rMaSp-based biomaterials to have desired property include additives or linkers that recognize cell binding site, or are targeted to the affinity binding domains (Bini et al., 2006; Widhe et al., 2013; Jansson et al., 2014) that improve physical properties (Teulé et al., 2012), and that control tertiary structures through phosphorylation and dephosphorylation (Winkler et al., 2000).

Although not specifically discussed in the literature, other potential challenges may include: 1. the accumulation of negatively charged rMaSp1 that may cause toxicity to the host cell, 2. the post-translational modification system in the host may not match that of the spiders, 3. lack of economic feasibility to obtain a desired yield of rMaSp, 4. challenges with optimization of the process of fiber assembly and biomaterials development.

Non-plant hosts that have been used to produce major ampullate spidroins include *E. coli* and *Salmonella* (Lewis et al., 1996; Arcidiacono et al., 1998; Widmaier et al., 2009; Xia et al., 2010; Edlund et al., 2018), yeast *Pichia* (Fahnestock and Bedzyk, 1997; Gaines and Marcotte, 2011; Liu et al., 2018), protozoa *Leishmania* (Lyda et al., 2017), mammalian cell lines (Lazaris et al., 2002), silkworm transformed with fusion protein or CRISPR/Cas9 site specific exchange (Miao et al., 2006; Teulé et al., 2011; Zhang et al., 2019), and mice or goats (Service, 2002; Xu et al., 2007). Here we focus on the efforts and strategies of recombinant spidroin production from plants.

#### Targeted Recombinant Spidroin Accumulation

**Figure 2:**
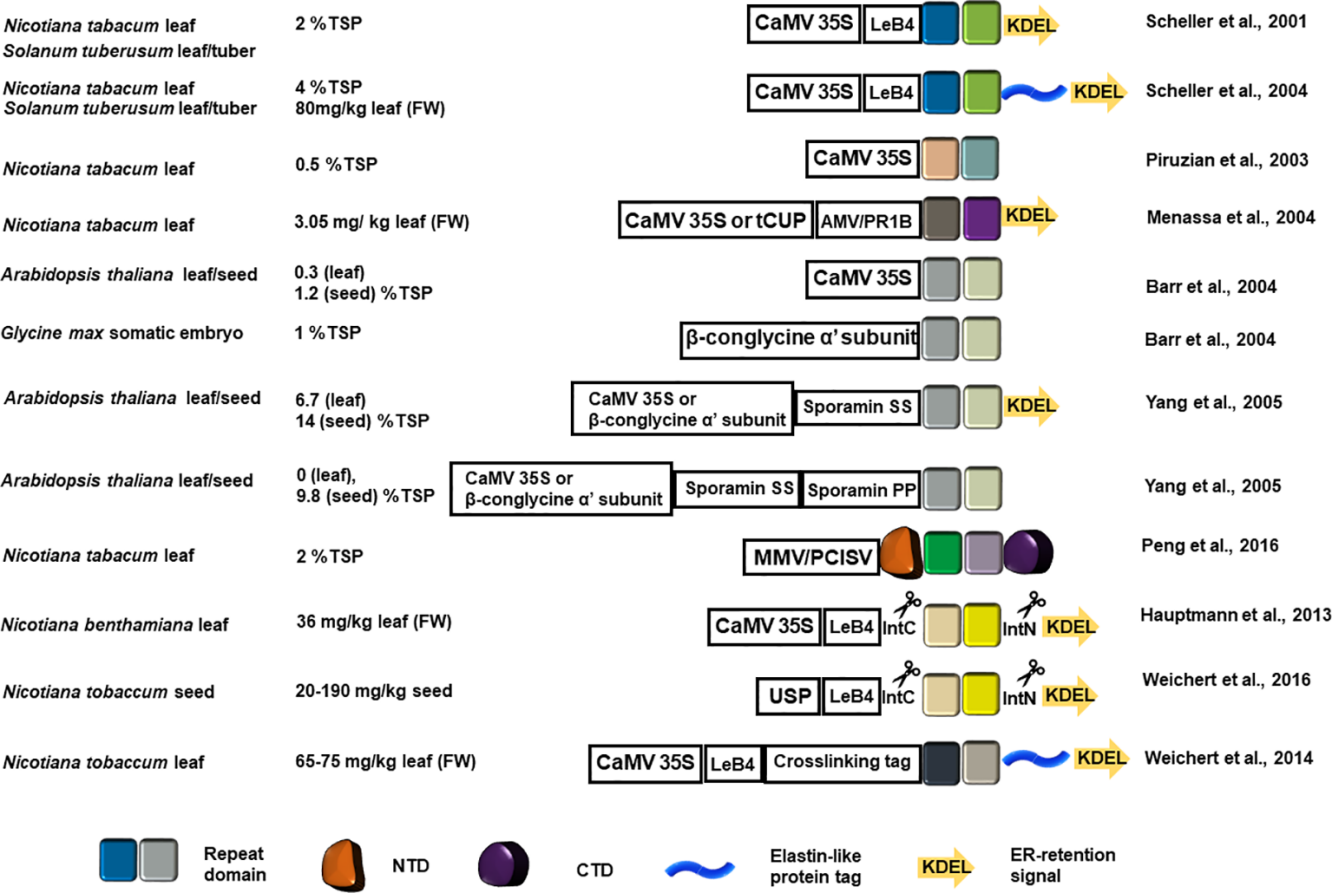
Schematic representation of the recombinant spidroin constructs introduced into a plant host system and the estimated yield. The colored squares represent the designed recombinant repeat blocks. Color variation indicates repeat block sequence variation in each application. The NTD and CTD are represented as in **Figure 1**. The elastin-like protein tag is shown as a blue helix and the ER retention signal is shown as a colored arrow. CaMV 35S, Cauliflower mosaic virus promoter; AMV, Alfalfa mosaic virus translational enhancer; PR1b, tobacco secretory signal peptide PR1b; MMV, Mirabilis mosaic virus full length promoter; PCISV, Peanut chlorotic streak caulimovirus full length promoter; USP, unknown seeds protein promoter; LeB4, LeB4 signal peptide; IntN, intein N-terminus; IntC, intein C-terminus.

Recombinant production localized at the endoplasmic reticulum (ER), using the ER retention signal KDEL at the C-terminus, can improve the yield of recombinant proteins as evidenced by the results summarized in **Figure 2**. Thermally stable MaSp1 repeat domain analogs (up to 100 kDa) with a C-terminal KDEL ER retention signal accumulated up to 2% of total soluble protein (TSP) in tobacco and potato leaf (Scheller et al., 2001), which demonstrated for the first time that rMaSp can be produced from plant host. The yield of rMaSp targeted to ER is four times higher as compared to the MaSp1 analogs 1f5 and 1f9 (fusion protein with a tetramer or octamer of the repeat domain) that accumulated in tobacco leaf without an ER target signal (0.5% of TSP) (Piruzian et al., 2003). MaSp2 and MaSp1/MaSp2 analog rADF-3 (containing the repeat motifs ASAAAAAA, GPGGQGPYGPG, GGYGPGS, and (GPGQQ)_n_) were also targeted to the ER (using KDEL) and produced in tobacco leaves (Menassa et al., 2004). The maximum production yield for ER-directed MaSp1 and MaSp2 analogs were 0.68 and 3.05 mg/kg fresh leaf tissue, respectively. The production of spidroin-like proteins was successfully retained when stably-transformed tobacco plants were transferred into the fields.

MaSp1 synthetic analogues (8 or 16 copies of DP1B monomer sequence GQGGYGGLGSQGAGRGGLGGQGAGA_7_GGA) were transformed into *Arabidopsis* using the cauliflower mosaic virus (CaMV) 35S promoter for leaf expression and the β-conglycine α' subunit promoter for *Arabidopsis* seed and somatic soybean embryo expression. Although *Arabidopsis* seeds showed better recombinant spidroin recovery, soybean embryos produced lesser amounts of the 127 kDa DP1B 16-mer (Barr et al., 2004), indicating that the length of the recombinant spidroin repeat domain affects tissue specific production yield. Yang and colleagues compared the yield of the same synthetic MaSp1 analogs targeted to apoplast, ER lumen, and vacuole in *Arabidopsis* leaf or seeds using sporamin-targeting determinant peptides and the ER retention KDEL peptide. Transgenic plants with seed-specific ER targeting constructs produced the highest amount of recombinant spidroins (18% TSP) (Yang et al., 2005).

#### Spidroin Mimics Design

The sequences of the rMaSp in the aforementioned efforts are derived from MaSp repeat domains. The importance of non-repetitive domains at the N-, and C-terminus in spidroin solubility and fiber assembly has propelled the inclusion of the terminal domains in synthetic constructs (Huemmerich et al., 2004a; Ittah et al., 2006; Hagn et al., 2011). In this context, mimetic spidroins with native N- and C-terminal domains flanking various numbers of consensus block repeat domains of MaSp1 or MaSp2 were produced in tobacco leaves (Peng et al., 2016). For MaSp1, constructs with 8 and 16 copies of repeat domains with flanking terminal domains were detected from tobacco leaf extracts and the yield for rMaSp1R8 (8 copies of repeats) was 0.7% TSP. For MaSp2, 8, 16, and 32 copies of the repeat domains with flanking terminal domains were detected, and the yield of rMaSp2R8 (8 copies of repeats) was 2% TSP. Each rMaSp was represented as an intact full-length protein from the crude leaf extracts despite some autonomous removal of the C-terminal intein tag of rMaSp2. The N- and C-terminal domains substantially increased the solubility of the rMaSp and allowed retention of the concentrated rMaSp in a liquid state even after freeze-drying (Peng et al., 2016).

#### Elastin-Like Polypeptide Tags That Facilitate Protein Purification and Protein Body Formation

Fusing the MaSp1 repeat domain mimic protein with 100 copies of VPGXG (X= G, V, or A) elastin-like polypeptide (ELP) tag led to a high yield (80 mg/kg leaf) when the fusion protein was directed to the ER of tobacco and potato leaves (Scheller et al., 2004). The solubility of ELPs is temperature-dependent and at temperatures below the melting temperature rMaSp1-100xELP fusion protein is insoluble and, therefore, isolable from other soluble proteins through precipitation. Through an inverse transition cycling to increase the temperature, the rMaSp1-100xELP fusion can be reconstituted into the buffer (Meyer and Chilkoti, 1999). ELP also induces protein bodies that sheath the recombinant proteins, leading to increased recombinant protein accumulation (Conley et al., 2009a).

#### Post-Translational Modification (PTM) and *In Vitro* Modification

Plants, as eukaryotic hosts, can provide PTM as recombinant proteins mature *in vivo*. However, the PTMs equivalent to those described in silk glands have not been reported from spidroins produced from plants. This may result from either the lack of PTM analysis being performed or the PTM of plant-derived spidroin is undetectable due to low efficiency of the equivalent plant enzymes. With the variation of number of domains designed and the sequence variation within domains, the PTM patterns may be different from each study.

Plant hosts have been shown to process an N-terminal precursor peptide, the legumin type B precursor signal peptide (LeB4), from MaSp1 repeat domain analogs produced in tobacco leaves and potato leaves (Scheller et al., 2001).

Intein-mediated PTM has facilitated repeat domain elongation and produced rMaSps with large numbers of repeat domains, some reaching the size of native spidroins. A flagelliform (FLAG) spidron flanked by intein self-splicing elements can be directed to the ER in tobacco leaves. After translation, intein self-splicing, and end joining, FLAG proteins of various sizes were produced (Hauptmann et al., 2013). Synthetic FLAG spidroin sequence, flanked by intein splicing elements and directed to the ER in tobacco seeds, produced multimers of up to 450 kDa with an estimated yield of 20–190 mg per kg seed (fresh weight). The recombinant FLAG proteins produced in tobacco seeds are stable at 15°C for one year (Weichert et al., 2016). Another example of *in vitro* PTM is multimerization of rMaSp1-100x ELP fusion protein tagged with lysine or glutamine by transglutaminase, which can produce near native sized spidroin-like recombinant protein (Weichert et al., 2014).

### Plant-Derived Spidroin-Based Biomaterials and Their Applications

Spidroin analogue-based materials are hypoallergenic, non-toxic, non-hemolytic, and minimally induce inflammatory reactions (Gomes et al., 2011; Dams-Kozlowska et al., 2013; Thurber et al., 2015; Hauptmann et al., 2015; Kuhbier et al., 2017). Therefore, their potential use in biomedical application is propitious and emerging. To be applied biomedically, spidroin analogue-based fibers, or materials can be autoclaved in water. The conformation or cytotoxicity of the materials does not change by sterilization (Lucke et al., 2015). Spidroin analogue-based materials can also be sterilized through ultra-violet radiation (Hafner et al., 2017), although UV may reduce the strength of the fibers (Lai and Goh, 2015). Here, we describe biomaterial formation from plant-derived spidroin mimics and their potentials in regenerative medicine and other applications.

#### Regenerative Medicine

Coatings derived from rMaSp1(repeats)-100x ELP fusion protein produced in tobacco leaves led to increased human chondrocyte cell growth and cellular mass. The overall stimulatory effect was equivalent to that observed with collagen coatings and twice as much as the non-coated control plate (Scheller et al., 2004). Collagen coating also induced long fibroblastoid morphology, which is disfavored in chondrocyte culturing. On rMaSp1-100x ELP coating, however, the chondrocyte cells are round-shaped similar to cells found *in vivo*. Coatings of similar rMaSp1(repeats)-100x ELP fusion protein produced from tobacco leaves formed polymer films. Coatings of these proteins significantly stimulated murine embryonic fibroblast cells after 24 hours of incubation, an indication of cell proliferation (Hauptmann et al., 2015). In addition, hydrogels made from tobacco-derived rMaSp1R8 (N- and C-termini flanking eight copies of repeats) and rMaSp2R8 (N- and C-termini flanking 8 copies of repeats) promoted human dental pulp stem cell attachment and proliferation (Hafner et al., 2017).

#### Diabetes

Recombinant spidroin-like proteins are stable under normal seed storage conditions (Weichert et al., 2016). An economic advantage of transgenic crop seeds producing recombinant protein for medicinal usage is that subjects can be fed directly thereby bypassing cumbersome protein purification. Transgenic rice producing *Araneus ventricosus* MaSp repeats and C-terminus was used to feed diabetic BKS.Cg-m+/+Lepr^db^ mice. The transgenic rMaSp-producing rice lowered the blood glucose of the diabetic mice. While the mechanism(s) by which *A. ventricosus* rMaSp lowers blood glucose in this animal model is in question, the study reports differences in cellular localization of receptor substrate 1 (IRS1), six-transmembrane protein of prostate 2 (STAMP2), and adenosine monophosphate-activated protein kinase (AMPK) (Park et al., 2019).

## Collagen-Based Scaffold Proteins Produced From Plants

### Collagen Sequence, Structure, and Folding

**Figure 3:**
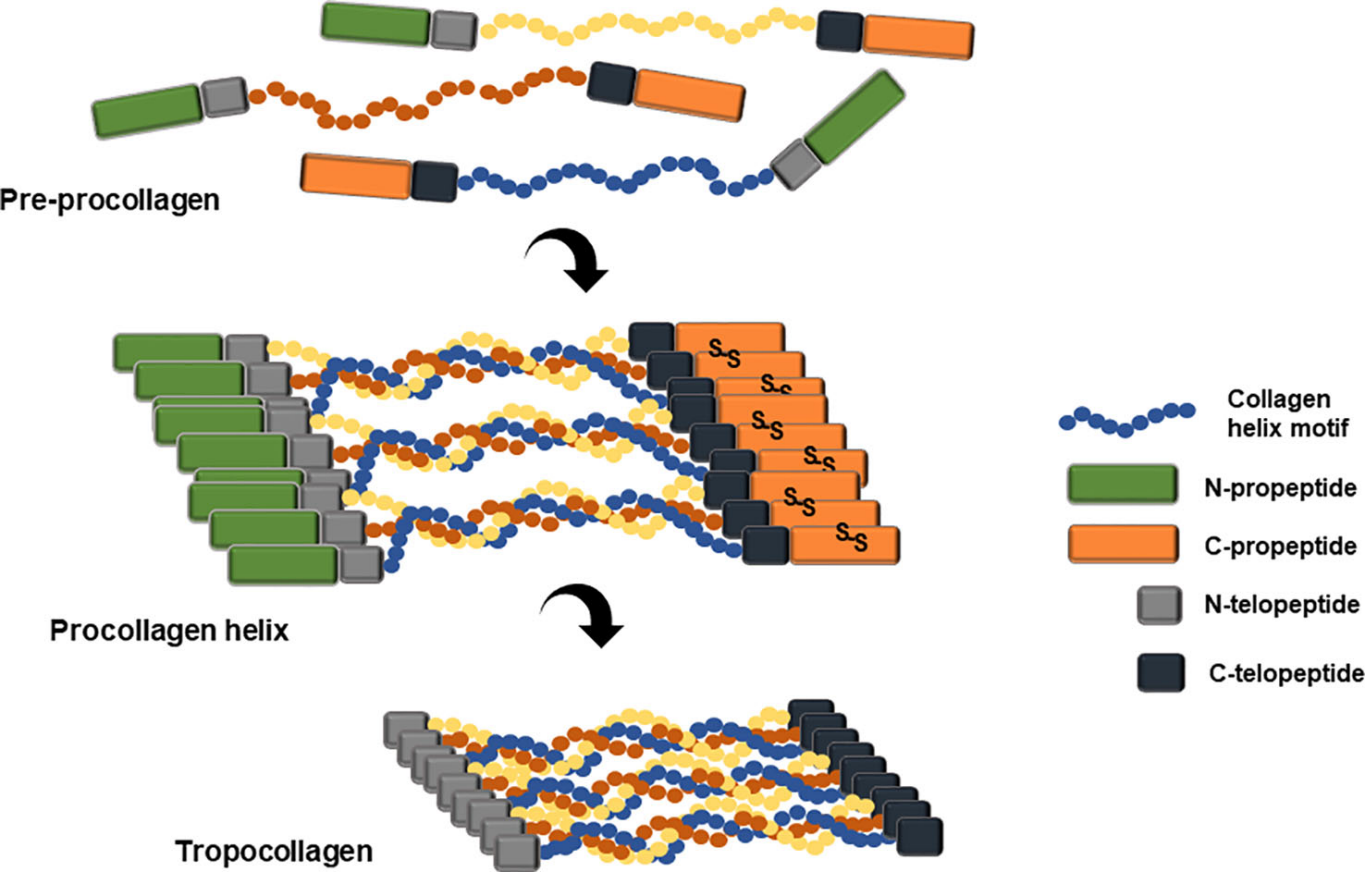
Schematic representation of pre-procollagen structure and the formation of tropocollagen. Pre-procollagen is translated and post-translationally modified by various enzymes including the prolyl hydroxylase and lysyl hydroxylase. The mature procollagen initiates triple helix formation through C-propeptide alignment and disulfide bond formation and twists along the helix motifs reach the N-propeptide. Several triple helix chains clusters align with each other and the N- and C- propeptide are cleaved by the N- and C- proteinase, either intracellularly or extracellularly, resulting in newly formed N- and C-termini, named N- and C-telopeptide. This tropocollagen helix stacks and packs into staggered collagen fibrils and alternately forms collagen fibers through multiple crosslinking reactions.

Collagen is a primary extracellular matrix scaffold protein of many tissues including connective tissues, basement membrane, skin, vascular tissue, brain, and spinal cord (Miller and Gay, 1982). Collagen biosynthesis is initiated from its precursor protein, procollagen. Variation in sequence, chain length, and chain combination leads to 29 different human collagen molecules (Sorushanova et al., 2019). Post-translational modification of procollagen chain includes lysyl and prolyl hydroxylation by lysyl hydroxylase (LH) and prolyl 4-hydroxylase (P4H) (Hutton et al., 1967; Kivirikko et al., 1992; Myllyharju, 2003). The collagenous region of the procollagen chain is essentially composed of repetitive motif, Gly-Xaa-Yaa, where Xaa is proline and Yaa is hydroxyproline. The procollagen molecule has flanking non-collagenous N- and C-terminal propeptides, which play critical roles in chain registration and helix formation through a zipper-like propagation starting from the C-propeptide trimerization of disulfide bond (**Figure 3**; Kirk et al., 1987; Engel and Prockop, 1991).

Three collagen monomer chains assemble into a triple alpha helical fibril, as a homotrimer or a heterotrimer (Brodsky and Ramshaw, 1997). The flanking N- and C- propeptides are cleaved by the procollagen N- and C-proteinases and the newly formed N- C-terminus are denoted as the N- and C-telopeptides. The resulting tropocollagen self-assembles into collagen microfibrils through crosslinking events mediated by lysyl oxidase and transglutaminase or through non-enzymatic glycation (Koide and Nagata, 2005). The collagen microfibrils assemble into collagen fiber with a characteristic D-periodic striation structure (Shoulders and Raines, 2009). Hydroxyproline composition of a non-recombinant human type I collagen homotrimer is 10.8%, and 10.3% for non-recombinant human type I collagen heterotrimer (Nokelainen et al., 2001). Hydroxyproline is critical for triple helix stability (Kotch et al., 2008).

### Plant-Derived Collagen

Although collagen is ubiquitously present in Animalia, the amino acid sequences and the physical properties vary among species (Stover and Verrelli, 2010). Collagen can be obtained from animal tissues, such as bovine, fish, or mouse but collagen from animal sources causes allergic reaction and sometimes may contain pathogen contamination (Mullins et al., 1996).

Recombinant collagen has been produced in the yeast *Pichia pastoris* (Nokelainen et al., 2001; Olsen et al., 2005), *Saccharomyces cerevisiae* (Toman et al., 2000), mammalian cells (Geddis and Prockop, 1993; Fukuda et al., 1997; Frischholz et al., 1998), mammals (mouse, John et al., 1999), and silkworm (Tomita et al., 2003) with some success but yield is often limited and/or the recombinant proteins lack post-translational prolyl and lysyl hydroxylation. Although plant hosts produce an indigenous prolyl 4-hydroxylase (P4H), its specificity and affinity to collagen Xaa-Yaa-Gly motif are different than the animal P4H (Tanaka et al., 1981; Hieta and Myllyharju, 2002). For stable triple helix assembly of plant-derived collagen, co-transformation of human P4H is required.

#### ER-Targeted Recombinant Collagen Accumulation

**Figure 4:**
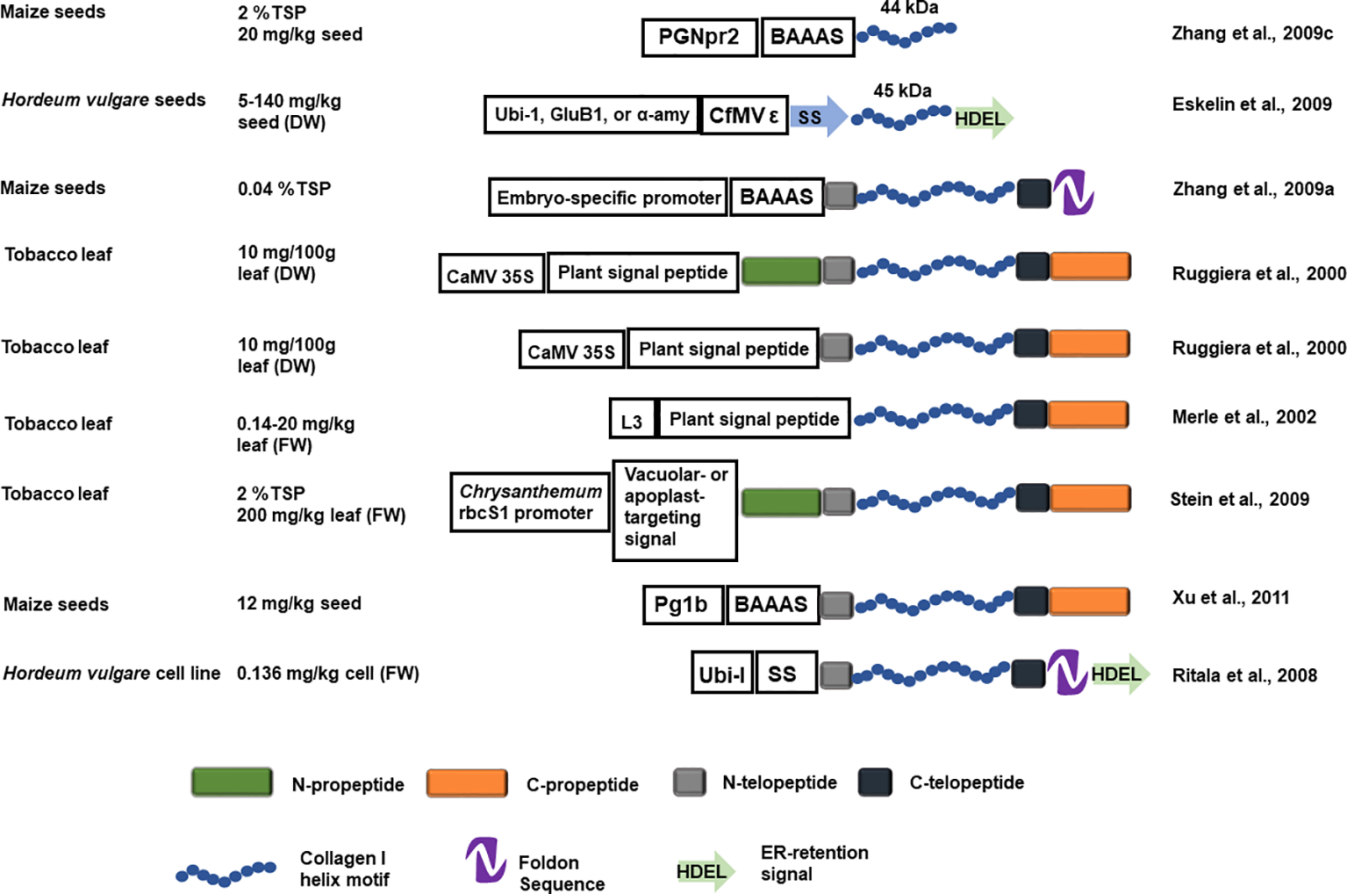
Schematic representation of the recombinant collagen constructs introduced into a plant host system and the estimated yield. The blue helix represents the designed recombinant collagen helix block, the N- and C- propeptide is represented by green and orange rectangles. The N- and C- telopeptide is represented with grey and black rectangles. The NTD and CTD are represented as in **Figure 1**. The foldon sequence is represented by a purple hairpin shape and a green arrow represents the ER-retention signal. GluB1, rice glutelin B1 promoter; α-amy, barley α -amylase fusion promoter 46 /4-6; BAAAS, Barley α -amylase signal sequence; Ubi-I, maize ubiquitin promoter and first intron; SS, Arabidopsis basic chitinase signal sequence; ϵ, the 5'UTR of Cocksfoot mottle virus; Pg1b, maize embryo specific globulin-1 promoter; PGNpr2, embryo-specific maize globulin-1 promoter; L3, L3 promoter.

A fusion construct of a human procollagen chain helical region, with flanking N- and C-telopeptide, the bacteriophage T4 fibritin foldon (a fusion polypeptide that is expected to facilitate helix assembly), and an ER-targeting signal, collectively called hCIα1, was transformed into barley. The stably transformed barley cells produced an ~130 kDa unprolylhydroxylated peptide (rhCIα1) up to 0.136 mg/kg (fresh weight). The rhCIα1, however, was unstable with a low melting temperature and no PTM phosphorylation was detected (**Figure 4**; Ritala et al., 2008). ER-targeted full-length rhCIα1 chain helical region, with flanking N- and C-telopeptides, is barely detectable in transgenic barley seeds (Eskelin et al., 2009). However, a 45 kDa abbreviated proα1(I) chain helical region, with flanking N- and C-telopeptides, can be produced in barley seeds. When expressed under the rice glutelin B1 promoter, the 45 kDa rhCIα1 accumulated to 140 mg/kg in T_1_ seeds, whereas the barley α-amylase fusion promoter 46/4-6 only directed expression of 2 mg/kg in T_1_ seeds. Doubled haploid (DH) progeny were generated and the 45-kDa rhCIα1production achieved from the best DH lines was 13 mg/kg dry seeds under the ubiquitin promoter and 45 mg/kg dry seeds under the glutelin promoter. Purified 45 kDa rhCIα1displayed only low levels of hydroxylated proline (2.8%, Eskelin et al., 2009).

Full-length proα1(I) chain, with the flanking N- and C-telopeptides and foldon, has also been produced in transgenic corn seeds after *Agrobacterium*-mediated transformation into corn immature embryos. The rhCIα1 yield is estimated to be 0.04% of TSP, with a low prolylhydroxylation rate (1.23%) and low melting temperature (26-27°C). A portion of the rhCIα1 is able to form triple alpha helices. Sequencing revealed that the N-terminal telopeptide had been removed but it remains unknown whether the removal occurred during *in vivo* protein maturation or during the protein purification steps (Zhang et al., 2009a). Protein purification optimization using this low yield rhCIα1-producing corn seeds was assessed after spiking with *Pichia*-produced proα1(I) chain with or without foldon. A combination of high purity without too much compromise on yield is achieved when the foldon is cleaved after the fusion protein is precipitated from the crude extract (Setina et al., 2015). While there is no difference in the rhCIα1 recovered from dry milling or wet milling of the transgenic corn seeds, dry milling may be preferred for practical reasons at the farm (Zhang et al., 2009b).

#### Collagen Mimics Design

Recombinant human procollagen proα1(I) chain (~120 kDa) with the flanking N- and C-terminal telopeptides plus propeptides, or the C-terminal telopeptide plus propeptide alone have been stably transformed into tobacco. Absence of the N-terminal propeptide led to production of truncated rhCIα1 protein of various lengths indicating it is required to maintain the integrity of full-length rhCIα1 during translation and protein extraction. Interestingly, the N-terminal propeptide of rhCIα1 was intact in plantlet extracts but removed from rhCIα1 found in mature tobacco leaf extracts. The C-terminal propeptide forms a disulfide bond in the precursor protein but C-terminal propeptides are cleaved both in plantlets and mature plants. The rhCIα1 is minimally prolylhydroxylated and unstable. However, alpha helices are formed and can be detected through circular dichroism spectra. Triple helix chain assembly may be facilitated by protein disulfide isomerase (Ruggiero et al., 2000). The above tobacco leaf-derived unhydroxylated collagen I (without N-terminal telopeptides) was used to determine the function of hydroxyproline in collagen folding and fibril formation. Hydroxyproline stabilizes the triple helix chain and assists a faster reassembly after denaturation. Unprolylhydroxylated collagen adopts more flexible conformation as compared to prolylhydroxylated collagen under the same temperature. Unprolylhydroxylated collagen fails to form the D-periodic striation in the fibrils at physiological ionic strength conditions but can form the D-periodic striation at lower ionic strength (Perret et al., 2001).

#### Post-Translational Modification (PTM) and *In Vitro* Modification

Human procollagen proα1(I) chain (with N- and C- telopeptides but without N- propeptide) has been produced in tobacco leaves through transient or stable transformation along with a chimeric proline-4-hydroxylase (P4H) consisting of a *Caenorhabditis elegans* α subunit and mouse P4H β subunit. Stable co-expression in this system yielded approximately 0.14-20 mg rhCIα1 per kg of leaf and 8.41% prolylhydroxylation. Transient co-expression led to 0.5–1 mg rhCIα1 per kg of leaf material and 6.84% prolylhydroxylation. The helical structure was assembled and the melting temperature (37°C) agreed with the level of prolylhydroxylation (Merle et al., 2002).

Transgenic plants containing human procollagen proα1(I) and proα2(I) chains constructs targeted to the vacuole, apoplast, or untargeted were obtained through co-transformation into tobacco. Only plants directing vacuole-targeted procollagen expression produced suitable protein products. An additional transgenic line created by co-transformation of human p4H α subunit, human p4H β subunit, and human lysyl hydroxylase 3 (LH3), that were also targeted to tobacco vacuole, was crossed with the procollagen-expressing line. The resulting heteromeric recombinant collagen-expressing line displayed PTM prolyl hydroxylation and lysyl hydroxylation, which was comparable to native human collagen. The harvested leaves yielded ~2% of TSP of rhCIα1, stable triple helical fibrils resistant to pepsin digestion were formed, and the characteristic D-periodic striation structures were observed (Stein et al., 2009).

Human procollagen proα1(I) coding sequence with or without the P4H α subunit and P4H β subunit fusion construct was transformed into corn. The production of the P4H α and β subunit caused the reduction of overall full-length rhCIa1 yield but resulted in 18.11% of hydroxyproline modification in recombinant collagen, which formed stable triple helices (Xu et al., 2011). Despite prolyl hydroxylation and lysyl hydroxylation, no phosphorylation was detected (Merle et al., 2002) and no glycosylation was detected of a 44 kDa rhCIα1 fragment from corn seeds (Zhang et al., 2009c).

### Plant-Derived Collagen-Based Biomaterials and Their Applications

Collagen from an animal source such as bovine collagen transplant is frequently associated with dermatomyositis (Cukier et al., 1993). Recombinant collagens are alternatives to be used in wound healing (Davison-Kotler et al., 2019), drug delivery (Olsen et al., 2003), and regenerative medicine (Werkmeister and Ramshaw, 2012). The rhCIα1 obtained from the patented production from transgenic tobacco formed stable triple helical fibrils and supported peripheral blood mononuclear cells (PBMNCs) proliferation, comparable to human non-recombinant collagen type I (Stein et al., 2009). The same tobacco-derived collagens promote the attachment and the multiplication of endothelial, fibroblast, and keratinocyte cells (Willard et al., 2013). The fibroblast infiltration, epidermal differentiation, and cellular metabolism of engineered skin based on tobacco-derived collagen are equivalent to engineered skin supported by human and animal collagen source. However, the interleukin 1 beta (IL-1β) accumulation of the macrophage cells (THP-1) is significantly less evident on engineered skin containing tobacco-derived collagen, indicating the hypoimmunogenity of the plant-derived collagen matrix (Willard et al., 2013).

## Elastin-Based Scaffold Protein Produced From Plants

Elastin is another fundamental extracellular matrix protein component that is found in connective tissues, vascular tissues, and basement membrane (Halper and Kjaer, 2014). As it is named, elastin is a protein elastomer with superior mechanical strain and has frequent demand in tissue regeneration practices (Urry et al., 2002; Daamen et al., 2007). This has sparked considerable interest in recombinant production of the major precursor protein tropoelastin. Tropoelastin is a secreted extracellular matrix protein with predicted alternating hydrophilic and hydrophobic regions (Wise and Weiss, 2009). The conserved C-terminal motif GRKRK binds to integrin α_v_β_3_.

The tandem repeats of elastin-like peptides VPGXG (X= G, V, or A) have been used as a fusion tag to assist recombinant protein purification and protein body induction in applications in recombinant spider silk protein production (Scheller et al., 2004; Conley et al., 2009b). Recombinant production of full-length tropoelastin has been just recently explored. A 2175 bp-ELN *orf* of tropoelastin was synthesized and transformed into tobacco plants through transient infiltration. A band corresponding to ~70 kDa was detected during electrophoresis of fresh leaf extracts using an antibody against tropoelastin. However, recombinant tropoelastin degraded into two smaller molecular weight peptides when leaf extracts were frozen (Abdelghani et al., 2015). As the instability of protein going through freeze thaw of the leaf tissues will create a problem for potential harvesting and storage, recombinant tropoelastin directed to seeds may be more suitable, since recombinant protein can be stably stored in the seeds (Weichert et al., 2016).

## Osteopontin-Based Protein Produced From Plants

Osteopontin is a negatively charged glycoprotein that is secreted to extracellular matrices from bone cells and mesenchymal stem cells (Reinholt et al., 1990; Scatena et al., 2007). The myriad of functions of osteopontin in the extracellular matrices include integrin binding through the RGD site (Yokosaki et al., 2005), binding to fibronectin (Mukherjee et al., 1995) and collagen (Chen et al., 1992), inducing cytokine and chronicle immune response (Lund et al., 2009), and regulatory roles in diabetes and obesity (Kahles et al., 2014). A human osteopontin (OPN) construct driven by the CaMV 35S promoter was transformed into *Nicotiana benthamiana* leaves and allowed plant-based production of a ~50 kDa osteopontin recognized by anti-osteopontin antibody. Recombinant OPN accumulated up to ~100 µg rhOPN per kg leaf mass. Tobacco-derived OPN promoted proliferation of human periodontal ligament stem cells and transcriptional increase of osteogenic differentiation and bone mineralization related genes, OSX, DMP1, and Wnt3, indicating that the plant-derived product had retained its biological activity (Rattanapisit et al., 2017).

## Emerging Tools for Recombinant Scaffold Protein Production in Plants

The considerable success of laboratory scale production of scaffold proteins using plants as a host system has been possible thanks to advances in molecular biology tools. However, any individual approach may not produce the same result for each extraction strategy due to the specific characteristics of the recombinant proteins being produced, diversity among plant hosts, and variation in host tissue types. For example, directing recombinant proteins into the plant vacuole can be a useful tool to store recombinant protein secluded from the cytoplasmic enzymes (Marin Viegas et al., 2016). While this method increases recombinant collagen yield (Stein et al., 2009), it results in no detectable yield of recombinant spider silk proteins (Yang et al., 2005). Therefore, there is no universal tool that fits all recombinant protein types, host systems, and host tissues. The selection is mostly empirical, and the outcomes are largely unpredictable.

### Protein Body Tag

Fusion tags like the Zein protein tag Zera ^®^, (PPPVHL)_8_ (Llop-Tous et al., 2010), hydrophobin-I (Reuter et al., 2016), and the elastin-like polypeptide (ELP), VPGXG (X= G, V, or A), can induce the formation of protein body-like organelles (PBs) in transgenic plants. In these PBs, membrane surrounds the protein body protecting the recombinant protein from proteolytic degradation. Thus, protein body tag constitutes a promising tool to enhance recombinant protein accumulation in plant tissues. The ability to purify recombinant ELP fusion through inverse transition cycling (ITC) ameliorates the expensive chromatography steps (Meyer and Chilkoti, 1999). A total of 30 VPGXG repeats is sufficient for effective protein recovery through ITC (Conley et al., 2009b). ELP induces large protein bodies with ER-derived membrane secluding the recombinant protein, which remains within the ER instead of being permanently directed to the vacuolar storage. Interestingly, the fusion tags are not a strict requirement for PB formation and when the ER-directed recombinant protein concentration reaches 0.2% TSP, PBs can be formed (Saberianfar et al., 2015). The ability to induce PB formation has been used to increase expression of desirable recombinant proteins such as erythropoietin (EPO) and the human cytokine interleukin-10 (hIL-10) as these co-expressed recombinant proteins are passively sequestered to the interior of the PBs.

### GAANTRY System

Recombinant production of scaffold proteins is hindered by a lack of tools to assemble large numbers of tandem repeats and by difficulties in co-transformation of PTM enzymes to achieve the optimal mechanical property and physiological function of the product. The recently developed GAANTRY system (Collier et al., 2018) uses recombinase-facilitated excision of plasmid backbone sequence and allows gene stacking of up to 10 different modular sequences from different donor plasmids. The modularity can be different domains of the same gene, or enzymes with catalytic functions in the same pathway. To produce tobacco-derived recombinant collagens with the appropriate PTM, five different cloning processes, two co-transformations, and a subsequent breeding were needed (Stein et al., 2009). The GAANTRY system may minimize the number of transformations, the number of antibiotics needed for the selection, and could reduce the subsequent plant breeding steps. The cloning strategies recently developed for long repeat sequences (Riet et al., 2017) and the recursive directional ligation approach (Dinjaski et al., 2018) may be used to create donor plasmids for the GAANTRY system. Combinations of different sequence modules from the donor plasmid will essentially allow an array of molecules, each with a unique combination of domains.

## Potential Application of Scaffold Proteins for Drug Delivery and Drug Discovery

Recombinant scaffold proteins produced from plant hosts promoted cell attachment and proliferation (Stein et al., 2009; Hauptmann et al., 2015). With the known affinity of spider silk analogues for liposome binding and encapsulation of low molecular weight compounds (Antonenko et al., 2010; Hardy et al., 2013; Agostini et al., 2015), plant derived spider silk proteins may also be used for controllable intake and release of therapeutic molecules (Doblhofer and Scheibel, 2015). The special affinity of spider silk mimics to HER2 expressing cells (Florczak et al., 2014) and the tumor-homing peptides (Numata et al., 2011) provides opportunities for the spider silk-based materials to be used in targeted melanoma cell delivery.

In addition to providing a supporting scaffold, extracellular matrix proteins (fibronectin, laminin, and collagen) serve as protection barriers and mediators of signal transduction (Singh et al., 2012). Exposed or partially degraded ECM proteins are known for attracting pathogens and providing scaffold for pathogen proliferation (Steukers et al., 2012). Agglutinin-like adhesin from common fungal pathogen *Candida albicans* specifically binds to fibronectin, laminin, and collagen IV (Gaur and Klotz, 1997). The direct connection of fungal pathogenicity with extracellular matrix protein interaction is postulated based on pathogenic fungi *Paracoccidioides brasiliensis*; when cells were pre-coated with ECM protein laminin, the infection consisting of granulomas became more severe (Vicentini et al., 1994). Although extracellular matrix proteins have gained attention in drug discovery, no antifungal agents are designed targeting the interaction of fungal pathogen with host extracellular matrix proteins (Järveläinen et al., 2009). Currently, no known fabricated extracellular matrices have assisted the screen of antifungals. Fabrication of extracellular matrices from plant-derived collagen, tropoelastin, and osteopontin is possible, and have a potential in future antifungal discovery.

## Concluding Remarks

### Economical Feasibility

Despite the success in proof of concept demonstration of a variety of scaffold proteins produced in plants, the commercialization of plant-derived scaffold proteins has been limited (Schillberg et al., 2019). The feasibility and the success of utilizing biotechnology tools vary depending on specific plant type and plant tissues and is rather unpredictable. Transient infiltration production flow can be improved to reduce the cost using ready-to-use cryo-stock of *Agrobacterium tumefaciens* (Spiegel et al., 2019). The yield based on current methods ranges from microgram to about 200 milligram of recombinant protein per kilogram of leaf or seed tissues. The pilot scale purification of MaSp1-ELP fusion proteins from stable transformed tobacco leaves using heat and acetone precipitation followed by centrifugal inverse transition cycling, achieved 80 mg MaSp1-ELP per kg of leaves (Heppner et al., 2016). This yield still does not meet the commercially acceptable level (1–5 g recombinant protein/kg of plant tissue) (Zhang et al., 2009c). Revolutionary recombinant protein production methods and strategies are essential to allow industrial scale production. Other than prolyl hydroxylation detection of recombinant collagen, other PTMs and the regulation of the plant-derived scaffold proteins have not been explored.

### Biomaterial Assembly and Application

Biomaterials assembled from recombinant fibrous protein or extracellular matrix proteins have positively enhanced biomedical applications such as boosting pancreatic islets survival and promote human fibroblast and human dermal microvascular endothelial cell adherence and multiplication (Annabi et al., 2013; Johansson et al., 2015; Widhe et al., 2016; Pereira et al., 2017; Tasiopoulos et al., 2018). Fusion of scaffold proteins with antimicrobial peptides can inhibit bacterial infection (Gomes et al., 2011). Recent *N. clavipes* genome assembly revealed novel spidroin motifs, which can be engineered potentially and add to the complexity of the plethora of recombinant spidroin molecules. Recombinant collagen production can also be expanded to the other types of collagen, especially collagen type IV, which is the major component of basement membrane. The combination of spider silk analogues with the motifs from the extracellular matrix proteins collagen, fibronectin, and laminin may provide unlimited opportunities for functionalized biomaterials. Crosslinking methods such as click chemistry (Harvey et al., 2017) and photocrosslinking (Johansson et al., 2015) are also applicable to plant-derived scaffold proteins. These scaffold proteins have potentials to be developed into bioink for precise and consistent biomaterial fabrication (Xiao et al., 2011; DeSimone et al., 2017; Chawla et al., 2018).

## Author Contributions

CP and WM wrote the manuscript. LK provided helpful suggestions, provided critical comments, and helped edit the manuscript.

## Funding

This work is supported by the Clemson University Libraries Open Access Publishing Fund. LK and CP are supported by the NIH grant # 1P20GM109094-01A1. This project was also funded in part by Clemson University’s R-Initiative Program to WM.

## Conflict of Interest

The authors declare that the research was conducted in the absence of any commercial or financial relationships that could be construed as a potential conflict of interest.
