# Corrigendum: Advances in Plant-Derived Scaffold Proteins

**DOI:** 10.3389/fpls.2021.775296

**Published:** 2021-10-08

**Authors:** Congyue Annie Peng, Lukasz Kozubowski, William R. Marcotte Jr

**Affiliations:** Department of Genetics and Biochemistry, Clemson University, Clemson, SC, United States

**Keywords:** spider silk, collagen, elastin, scaffold, regenerative medicine, extracellular matrix

In the original article, the authors neglected to include that this project was funded in part by Clemson University's R-Initiative Program to William R. Marcotte Jr.

The authors apologize for this error and state that this does not change the scientific conclusions of the article in any way. The original article has been updated.

## Publisher's Note

All claims expressed in this article are solely those of the authors and do not necessarily represent those of their affiliated organizations, or those of the publisher, the editors and the reviewers. Any product that may be evaluated in this article, or claim that may be made by its manufacturer, is not guaranteed or endorsed by the publisher.

